# A Study of Physicochemical Properties of Stockpile and Ponded Coal Ash

**DOI:** 10.3390/ma15103653

**Published:** 2022-05-20

**Authors:** Rostislav Šulc, Martina Šídlová, Petr Formáček, Roman Snop, František Škvára, Adéla Polonská

**Affiliations:** 1 Department of Building Technology, Faculty of Civil Engineering, Czech Technical University in Prague, Jugoslávských Partyzánů 1580/3, 160 00 Prague, Czech Republic; petr.formacek@fsv.cvut.cz; 2 Institute of Glass and Ceramics, Faculty of Chemical Technology, University of Chemistry and Technology Prague, Technicka 5, 166 28 Prague, Czech Republic; martina.sidlova@vscht.cz (M.Š.); skvaraf@vscht.cz (F.Š.); adela.polonska@vscht.cz (A.P.); 3 ČEZ Energetické Produkty, s.r.o., Komenského 534, 253 01 Hostivice, Czech Republic; roman.snop@cez.cz

**Keywords:** stockpile ash, ponded ash, recovery, CCPs, physicochemical properties, coal combustion

## Abstract

The article describes chemical and also selected physical properties of ponded high temperature fly ash (FA) and bottom ash (BA) from a Mělník lignite power plant located in the Czech Republic. The research was carried out on samples obtained from drills with a depth of up to 54 m and the age of the samples retrieved from the lowest layers of the stockpile dating back to 1960. At the same time, a comparison was made with fresh fly ash and fresh bottom ash obtained from the identical power plant. The study analyzed a total of 98 stockpile samples. The properties selected were studied across the entire stockpile, namely moisture content, specific density, specific surface, carbon content, elemental and phase composition, pH, electrical conductivity and leachability. SEM analyses were also performed. The performed measurements of chemical properties proved the chemical stability of the material even after several decades of storage in the stockpile. The largest changes are evident in the results of the analyses related to the leachability of SO_3_, Cl^−^ and F^−^. In contrast, the pH does not change significantly, and the composition is pH neutral or alkaline. Regarding ponded BA, particle disintegration was noted depending on the increasing core borehole depth.

## 1. Introduction

The European Coal Combustion Products Association (ECOBA) states that in 2016, 28 EU countries produced over 105 million tons of coal combustion products (CCPs). The Czech Republic, while being a relatively small country, still manages to produce around 13 million tons of CCPs per year, which is primarily caused by a high share of non-combustible components in the coal burned. CCPs include fly ash (FA), bottom ash (BA), boiler slag, FBC-ash, FGD-gypsum and SDA-products. Generally, fly ash is formed by burning pulverized coal in coal-fired power plants, most often at temperatures of 1100–1500 °C [1]. FA is transported with a flue gas and captured on electrostatic precipitators or fabric filters. FA particles are mostly spherical, but they can also be of irregular or angular shapes. During combustion, bottom ash is formed consisting of coarse granular particles and is collected at the bottom of coal furnaces [2]. The particles are porous, irregular and rough-textured [3]. FBC-ash, on the other hand, is formed during fluidized bed combustion at significantly lower temperatures not exceeding 950 °C [4]. The particles of this type of ash are irregular and porous, and their morphology resembles the shape of the original coal grains. If limestone is additionally fed to the boiler to aid desulfurization, the resulting FBC-ash also contains anhydrite II and lime [5]. The valuable use of CCPs is primarily concentrated on the construction industry. The largest share of all CCPs produced consists of FA, which is subsequently employed in the widest range of applications, namely in the building and construction industry, civil engineering and for mine back filling [6,7]. Another use is offered by agriculture [8,9]. In addition, research is focused on alternative uses of FA such as the adsorption of SO_3_ [10], NO_x_ and Hg [11]. Bottom ash finds its application as a fine aggregate [12], highway material [13] or possibly for the production of bricks, fire-proof products, ceramics, etc. [14]. The use of FBC-ash is significantly more complicated compared to that of FA. However, it turns out that even this type of CCP may find a significant application in construction works [15,16].

A large part of the produced FA and BA has been deposited in stockpiles for decades, and their number is still increasing, despite the fact that today, not only in Europe, there is already a shortage of such materials on the market. With the closure of coal-fired power plants, this commodity will become increasingly inaccessible. It is therefore obvious that the reuse of fly ash from stockpiles is a pressing issue nowadays. In recent decades, analyses of fly ash have been carried out dealing with the ash deposited in stockpiles in the United Kingdom, the United States and, more recently, China. [1]. It turned out that each stockpile was unique, and an individual approach was needed for the efficient use of the extracted ash. It is evident that the research carried out in individual localities/countries will, in the future, contribute to a faster and more efficient use of the CCPs deposited worldwide.

Originally, ash was stored by flowing it to ponds in a dilute slurry [1]. As the ash flowed, it sorted and stratified, resulting in a heterogenous body of material. Currently, ash is mostly stored dry. The stockpile ash deposited in this way may be relatively homogeneous, but it has also been found that the agglomeration of fly ash particles can occur, especially in fly ash with a high free lime content [17]. In addition, when stored in storage facilities, fly ash comes into contact with water that is either used to store the fly ash to prevent the production of dust or may come from rain. Rainwater can cause large leaks through the fly ash layers, which is also the reason why fly ash in the stockpile has high moisture even at depth. Robl et al. state that the agglomeration is greatest at a moisture content of 10–20%. Research of samples (wet stored fly ash) up to 18 months old [17,18] showed agglomeration of the samples with the presence of sulfate-based products on particles. The pozzolanic reactions were also noted. Sambor and Szymanek [19] analyzed almost 300 samples from the ash stockpile at a depth of 0–2.5 m exposed to the weather and found that the pH of the core borehole rose with its increasing depth, which resulted in alkaline reactions that promoted the leaching of chemical compounds into the stockpile. Several other works [20,21] indicate changes in the surface morphology of the deposited fly ash particles, namely the surface dissolution and the formation of new phases (secondary phases).

Detailed research concerning ponded fly ash in the Czech Republic has not been carried out yet. The aim of this article is to present and compare the chemical, mineralogical and selected physical properties of deposited FA and ponded BA depending on the length and method of their storage, both coming from the locality Panský les, which is a repository for CCPs from the power plant Mělník in the Czech Republic. The results of this work are intended to facilitate stockpile ash recovery.

## 2. Materials and Methods

### 2.1. Sampling

**Figure 1:**
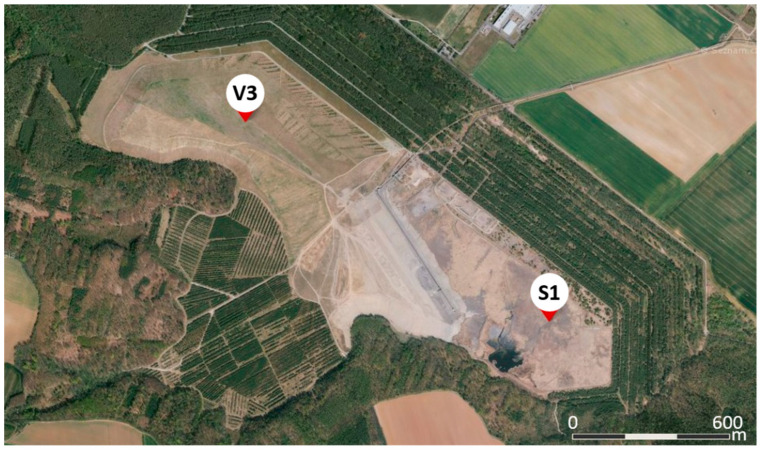
Location of core borehole V3 (50.4086175 N, 14.3813356 E) and S1 (50.4016497 N, 14.3977614 E) in stockpile Panský les, https://mapy.cz/ (accessed on 10 May 2022).

All samples were collected from the Mělník power plant located in the Czech Republic and from the stockpile and pond from the locality Panský les located next to the power plant. Stockpile Panský les is approx. 2200 m long, 550 m wide and from 60 to 25 m deep. The samples consisted of fresh fly ash (labelled FA) and fresh bottom ash (labeled BA) produced by the power plant (consumption in 2020). Furthermore, samples of deposited ash (stockpile or ponded ash, labelled V3) and ponded bottom ash BA (labelled S1) were collected as core borehole. The stockpile has been in operation since the 1960s. Until the 1990s, the ash had been ponded into local lagoons corresponding to the V3 core borehole depth of approx. 25 m in the form of a hydromixure. Since the early 1990s, fresh fly ash (FA) was transported from the Mělník power plant to the stockpile in the dry state. FA to be deposited used to be mixed with bottom ash (BA), the latter being at the amount of 5 to 15%. The greatest depth of 54 m was reached by core borehole V3. Samples from V3 core borehole depth exceeding 50 m contained subsoil. Furthermore, core borehole S1 was drilled in the segment of the stockpile, where only BA was ponded in the past. Core borehole S1 reached a depth of 44 m, with the last two meters, 43 m and 44 m, containing the limestone subsoil. All samples were collected by the Wirth B1A drilling rig. A total of 98 samples were acquired from both core boreholes V3 (54 samples) and S1 (44 samples), Figure 1. Approximately 10 kg of material were taken from each individual meter into a bucket and sealed so that no moisture escaped. The samples in the buckets were marked with a specific core borehole and depth, e.g., V3-5 m.

### 2.2. Methods

Moisture content of all samples from core boreholes V3 and S1 and from fresh FA and fresh BA was identified by drying the samples to the constant weight at 105 °C. Three samples of 200 g of material were taken from each drilled meter.

Loss on ignition (LOI) was performed at 950 ± 25 °C until reaching the constant weight, and each sample was tested 3 times.

The elemental composition of all ashes was expressed in oxides and determined by *X-ray fluorescence* (XRF) analysis. All samples were homogenized in a laboratory vibrating mill before measurement. For XRF analysis, ARL 9400 XP sequential WD-XRF spectrometer was used (Switzerland). The data obtained was assessed by the software Uniquant 4. Measurement errors were below 1 wt.%. All values were recalculated with respect to loss on ignition.

Diffraction patterns were collected at room temperature with an X’Pert^3^ Powder θ-θ powder diffractometer (PANalytical, Almelo, The Netherlands) with parafocusing Bragg-Brentano geometry using Cu Kα radiation (λ = 1.5418 Å, Ni filter, generator setting: 40 kV, 30 mA). An ultrafast PIXCEL detector was employed to obtain XRD data over the angular range from 15 to 75 ° (2θ) with a step size of 0.013 ° 2θ and a counting time of 180 s/step. The fixed divergence slit was applied for the measurement. The back loading technique was utilized to eliminate preferred orientation. The data were evaluated with software package HighScore Plus V4.6 (PANalytical, Almelo, The Netherlands) and search-match was performed in PDF4 + database. Subsequently, the Rietveld method was applied to calculate mass content for present crystalline phases and amorphous phase using ZnO (10 wt. 10%) as an internal standard. Measurement errors were below 3 wt.%.

SEM images were measured on a Hitachi S 4700 cold cathode scanning electron microscope (Tokyo, Japan) equipped with two secondary electron detectors and one reflected electron detector.

Leachability, pH and conductivity were determined from the analysis of extracts prepared by weighing 100 g of the dried sample with 1000 mL of distilled water, followed by homogenization for 24 h (EN 12457-4). The leachate was filtered through a 0.45 μm filter paper by the vacuum filtration system. The pH of the extracts was determined according to ČSN 10,523 using a pH meter 526 (WTW, GmbH & Co. KG, Weilheim, Germany) and conductivity according to ČSN EN 27,888 using a Cond Level 1 instrument (WTW, Czech Rep.). Furthermore, the amounts of chlorides, fluorides and sulfates were determined on an ion chromatograph IC 930 Compact Flex (METROHM, Herisau, Switzerland) according to ČSN EN 10304-1. Heavy metals were determined using a mass spectrometer ICP-NexION 300X (PERKIN ELMER, Waltham, MA, USA) according to ČSN EN 17294-1, 2. In addition, the amount of mercury was established according to ČSN 75 7440 using a single-purpose atomic absorption spectrometer AMA 254 (ALTEC, Chotěboř, Czech Republic). Measurement errors were below 1%.

Physical properties were monitored at all core boreholes and depths and included particle size distribution (PSD), specific density (ρ) and specific surface (S) analyses. A laser-light scattering analyzer Bettersizer (Bettersize Instruments Ltd., Dandong, Liaoning, China) was used to determine the particle size distribution. Specific density (ρ) was determined by the pycnometric method, and the specific surface (S) of the samples was measured by air permeability Blain method (ČSN EN 196-6).

Moisture content, PSD and loss on ignition analyses are presented in the article on all samples from core boreholes V3 and S1. For other analyses, representative samples were selected across the entire core borehole V3 and S1 to take into account all characteristics and material changes with increasing core borehole depth.

## 3. Results and Discussion

### 3.1. Moisture Content

**Figure 2:**
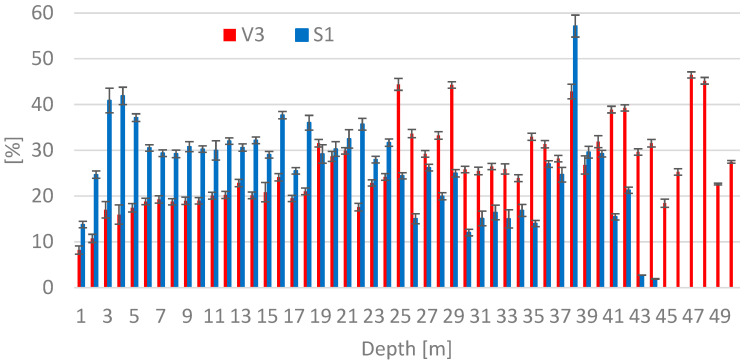
Moisture Values for Samples from Core Boreholes V3 and S1.

Determination of moisture in deposited ash V3 and bottom ash S1 is shown in the graph in Figure 2. The obtained results concerning moisture determination of deposited ash V from core borehole V3 are in the range of 17–47% at a depth of 3–50 m. At a core borehole depth of 1–2 m, the moisture value was 8%, resp. 11%, which was affected by the fact that sampling was carried out in the dry season of the year. In the case of bottom ash, the moisture values of the depths up to 24 m were higher, reaching about 30% in comparison with the V3 core borehole. In contrast, at the depth up to a maximum of 42 m, the moisture values were mostly lower in comparison with V3. An exception was the moisture of the sample from a depth of 38 m, where the highest value of 57.1% was recorded. The moisture content of the fresh ash analyzed was 6% while the bottom ash showed 23%.

### 3.2. Particle Size Distribution

**Figure 3:**
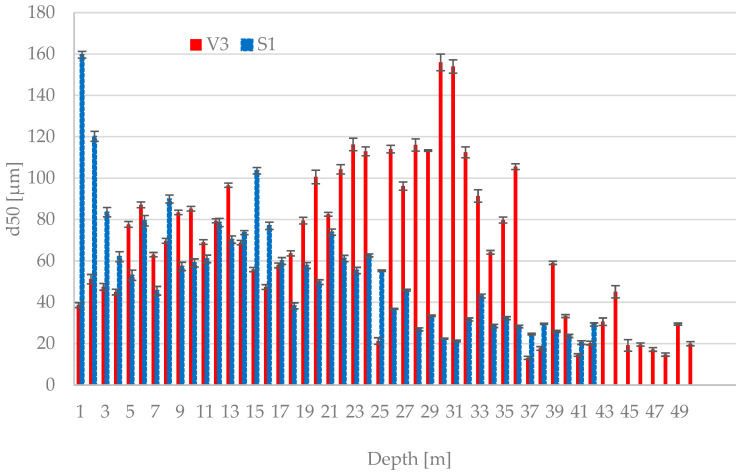
Mean Particle Size of Deposited Ash from Core Borehole V3 and Bottom Ash from Core Borehole S1.

Figure 3 shows the mean particle size for deposited ash from core borehole V3 and ponded BA S1. The mean particle size for fresh FA was detected at 36.2 µm, and for fresh BA, it was 124.8 µm.

The graphs show a relatively large variability in the mean particle size in core boreholes V3 and S1. The average values from all core borehole depths were 67 μm for core borehole V3 and 55 μm for S1. The highest value for core borehole V3 was recorded at the depth of 30 m, namely 156 μm, and the lowest value at the depth of 37 m, namely 13.1 μm. No significant trend across the core borehole was observed in V3. In contrast, the ponded BA from core borehole S1 shows a decreasing character of the mean particle size, and a comparison with the value of the mean particle size of fresh BA shows a significant disintegration of the particles over time. The disintegration of ponded ash particles was highlighted in the work of Robl et al. [1].

### 3.3. Specific Density and Specific Surface

Specific density (ρ) and specific surface area (S) were measured across the entire core borehole depth. The values for selected core borehole depths V3 and S1 are provided in Table 1. Specific density values ranged from 2029 to 2501 kg/m^3^ for core borehole V3 and 1912–2579 kg/m^3^ for core borehole S1. The average measured values were 2070 kg/m^3^ for core borehole V3 and 2222 kg/m^3^ for core borehole S1, which, in comparison with the value of fresh FA of 2296 kg/m^3^ and fresh BA being 2349 kg/m^3^, shows that fresh FA and BA have higher bulk density values on average compared to the deposited samples taken. The average value of the specific surface was 201 m^2^/kg for core borehole V3 and 180 m^2^/kg for core borehole S1. It can be seen here that the fresh samples had a higher specific surface area compared to the deposited samples, which is likely to be related to the agglomeration of the particles.

### 3.4. Carbon Content Measured as the LOI

**Figure 4:**
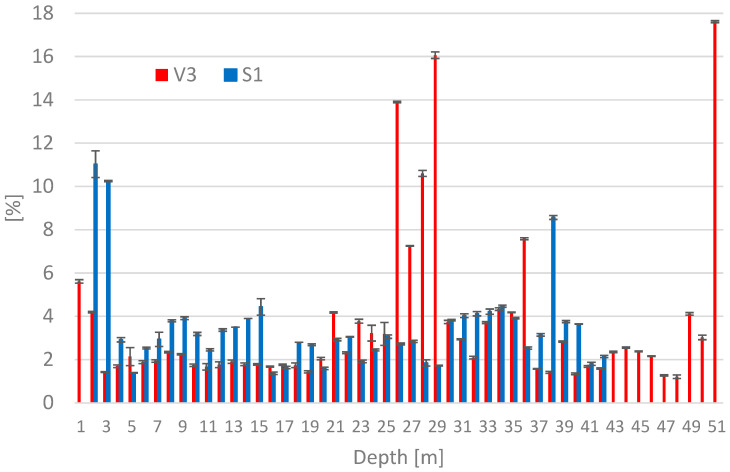
Determination of Loss on Ignition from Core Boreholes V3 and S1.

The amount of unburned carbon was determined by loss on ignition (LOI). Excessive amounts of carbon must be removed when using fly ash in cement, as it negatively affects the properties of the cement. Carbon is hydrophobic and, in addition, adversely affects the properties of plasticizing and aerating additives. The loss on ignition was determined to be 2.1% for fresh fly ash and 5.7% for fresh BA. The graph in Figure 4 shows the measured values of LOI for core boreholes V3 and S1. The carbon content of most samples did not exceed 5%, and according to ČSN EN 450-1, it is considered as low carbon content FA. The increased amount of unburned carbon 7.3–16.1 wt.% in core borehole V3 in the depth of 26–29 m is probably related to the change of the ash deposition procedure. The original wet method of ponding caused carbon particles to leach to the surface of the stockpile. When changing to dry storage, this layer was then covered with stockpile ash, which was only sprinkled with water. Dry storage of deposited ash was used until the stockpile was recultivated. The high value of unburned carbon content is probably related to the increased amount of moisture in the depth of 26–29 m in V3 core borehole.

### 3.5. Bulk Chemical Analysis

XRF analysis was used to perform the elemental analyses of fresh FA, fresh BA and deposited ash from core boreholes V3 (1–50 m) and S1 (1–42 m). Selected results of the analyses are presented in Table 2. 

By comparing fresh FA and BA with the samples of deposited materials, it can be stated that no significant changes were found in the composition of both the major and minor elements. The results show that the samples are rich in silica (SiO_2_), alumina (Al_2_O_3_) and iron oxide (Fe_2_O_3_). SiO_2_ content ranges from 47.48 to 61.95 wt.% for V3 and 45.58–61.98 wt.% for S1. The content of Al_2_O_3_ is in the range of 21.73–35.70 wt.% for V3 and 24.89–35.09 wt.% for S1. The Fe_2_O_3_ content ranges from 3.77 to 12.05 wt.% for V3 and 4.22–16.94 wt.% for core borehole S1. With respect to the use of fly ash in concrete, from the point of view of the ČSN EN 450-1 standard, it is required that the fly ash contains more than 70% of Al_2_O_3_, SiO2 and Fe_2_O_3_, and this requirement was met by all samples from core boreholes V3 and S1. The content of alkalis Na_2_O_eq_ (=Na_2_O + 0.658 K_2_O) is in the range 0.99–1.98 wt.% for V3 and 0.99–2.23 wt.% for S1. The alkali values meet the requirements of ČSN EN 450-1 for the total amount of alkalis, which must be less than 5 wt.%. Furthermore, the requirements for the maximum content of CaO, MgO and SO_3_ were also satisfied. The amount of CaO ranged from 1.57 to 2.88 wt.% for V3 and 1.51 to 3.88 wt.% for S1, with the exception of sample S1 38 m, where a CaO value of 13.08 wt.% was recorded. The high CaO value is not directly related to ponded BA. CaO in the form of CaCO_3_, Ca (OH)_2_ or CaO could be introduced into the stockpile as waste, or it could be introduced there in the form of a substrate for BA deposition in order to capture leached ions from the deposited material. The analyzed samples of fresh FA, BA and deposited ash are classified as siliceous ash (Class F) with a low CaO content < 5.1% and a content of primary oxides (SiO_2_ + Al_2_O_3_ + Fe_2_O_3_) ˃ 70%. The only dependence that can be observed was the changing sulfur content. The highest value detected in the fresh ash samples obtained was 0.56 wt.% of SO_3_. With an increasing depth of core borehole V3, this amount then decreases, which is probably related to leaching, with respect to the solubility of sulfates. At the same time, it is interesting to note that in the case of ponded BA, sulfur is present predominantly in the oxidation state of S^−2^ but occurring at a significantly lower concentration compared to the highest value detected in the sample of fresh BA. As for the ponded BA, its low sulfur content can be attributed to the wet deposition process, i.e., the faster dissolution of sulfates and the leakage through the bottom of the stockpile. From a chemical point of view, based on the results, both deposited ash and ponded BA can be classified as ash class F.

### 3.6. pH, Electrical Conductivity and TDS

The pH analysis shows that fresh FA had a pH of 8.23; see Table 3. In the case of the deposited ash from core borehole V3, pH was between 6.87 and 7.90. Here, the lowest value corresponds to 16 m and the highest value is related to 28 m. For core borehole S1, pH was 7.38 for fresh BA, and in the case of the bottom ash from core borehole S1, pH varied between 6.92 to 7.68. An exception is constituted by sample S1 from the depth of 38 m, where the pH value 10.39 was recorded. This highest value of pH (10.39) is related to the high amount of CaO detected with XRF analysis. In sum, the entire stockpile V3 and S1 is pH neutral or alkaline. At the same time, no significant changes of pH were detected in the samples from the S1 core borehole compared to fresh bottom ash. Weak changes in pH were detected for the V3 core borehole between fresh FA and deposited FA. The changes in pH could have occurred due to long-term exposure of deposited ashes in the stockpile to rainwater, as rainwater is generally acidic. Acidic solutions can affect the mobility and leaching of elements in FA, as described by Dutta et al. [22].

The value of electrical conductivity (EC) for the fresh FA sample was 260 mS.m^−1^, as shown in Table 3. The EC values for core boreholes V3 and S1 are significantly lower and range from 14.0 to 70.1 mS.m^−1^ for core borehole V3 and from 10.1 to 57.0 mS.m^−1^ for core borehole S1. Typically, high EC values in fresh fly ash indicate a higher content of ions in the leachate from the fly ash. In contrast, lower EC values indicate a significant ion loss in the stored material. This finding is an indicator of the ions leaching from deposited materials. These conclusions are consistent with the work of Bhattacharyya et al. [23] and Eze et al. [21], who studied weathered coal FA in South Africa.

Table 3 further shows the values of the total dissolved solids (TDS) detected in leachates. The highest values were again identified for fresh FA and BA, namely 2220 mg/L and 2430 mg/L, respectively. The values for core borehole V3 range from 444 mg/L up to 100 mg/L, while, for core borehole S1, all the values are below 122 mg/l except for sample S1 from 38 m, displaying a value of 388 mg/L. The TDS results are analogous to the EC results. At the same time, the results clearly demonstrate that the elements leached to a greater extent from core borehole S1 compared to core borehole V3. This finding is probably again related to the ponding of BA.

### 3.7. Leachability (Trace Elements)

The leachability results for trace elements with selected core borehole depths are presented in Table 4, including the limit values as set by the National Association for the Utilization of Energy Products (ASVEP) and based on the Waste Act 294/2005 Coll. The values that do not meet the ASVEP limits are highlighted in bold. The limit values for arsenic 0.01 mg/L, molybdenum 0.005 mg/L, selenium 0.01 mg/L and vanadium 0.018 mg/L were exceeded in almost all the samples provided. The limit values for barium (0.05 mg/L) and boron (0.3 mg/L) were also exceeded for both fresh FA and BA as for deposited samples, but, in this case, this phenomenon occurred only at smaller core borehole depths. The limit values for antimony, tin, chromium, cobalt, lead, copper, nickel and mercury were not exceeded in any of the samples; the values were below the detection limit of the instrument and are, therefore, not listed in the table. The leachate values for chlorides, fluorides and sulfates are provided below. When comparing fresh FA and BA samples with deposited samples, it is evident that some ions, especially sulfates, leach out. In general, the samples from smaller core borehole depths contained fewer ions.

### 3.8. XRD

**Figure 5:**
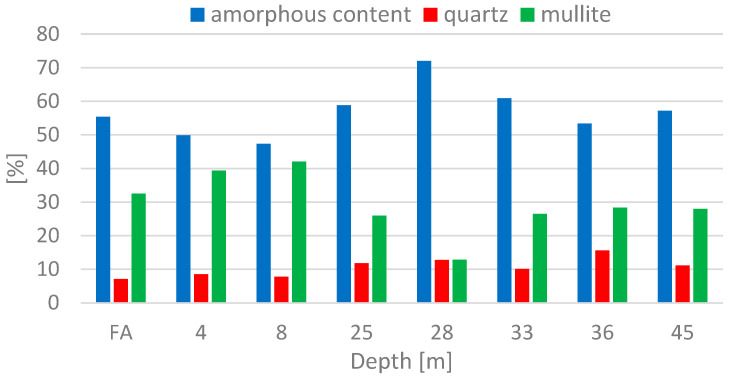
Representation of Major Phases Obtained by XRD for Fresh Fly Ash and Deposited Ash at Selected Depths from V3. Measurement errors were below 3 wt%.

**Figure 6:**
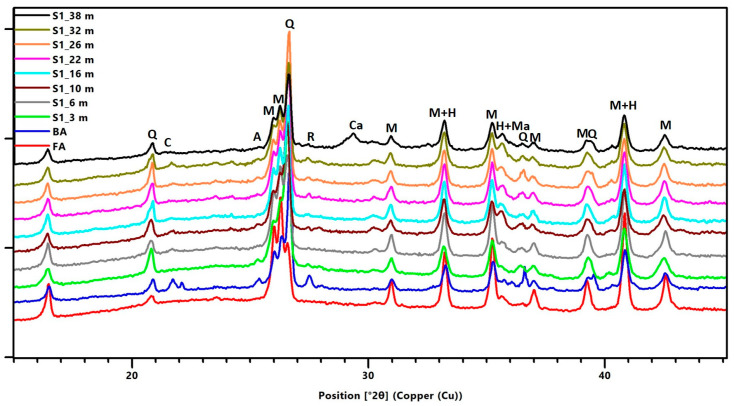
X-ray Diffraction Patterns of Fresh FA, Fresh Bottom Ash BA and Deposited Bottom Ash from Core Borehole S1; M-mullite (01-084-1205), Q-quartz (01-089-8934), Ca-calcite (01-078-4614), H-hematite (01-089-8103), Ma-magnetite (01-084-2782), A-anatase (01-089-4921), R-rutile (04-005-4674), C-cristobalite (04-007-2379).

The phase composition of fresh FA and samples taken from core borehole V3 from depths of 4, 8, 25, 28, 33, 36 and 45 m as determined by XRD analysis shows that the primary crystalline phases are mullite and quartz, while the minor phases are represented by hematite, magnetite, rutile, anatase, calcite and cristobalite. The amorphous phase was in the range of 47–72%; see Figure 5 and Figure 6. The highest value of the amorphous fraction recorded in the depth of 28 m is related to the great amount of unburned carbon, which was determined to be 11 wt.% for this particular depth by means of the loss on ignition. Fresh fly ash contained about 55–56% of the amorphous phase. No significant differences were detected in the phase composition of fresh FA and deposited ash. At the same time, no XRD did not identify any crystalline hydration products that could adversely affect the pozzolanic properties of deposited ash.

The phase composition of ponded BA for S1 in selected depth meters 3, 6, 10, 16, 22, 26, 32 and 38 did not differ significantly from the phase composition of the agglomerate, with respect to fresh fly ash and fresh bottom ash (BA). The following Figure 6 presents diffractograms for fresh FA, fresh BA and for selected meters of core borehole S1 with their main crystalline phases. The primary phases are again mullite and quartz. The diffractograms also show that the percentage representation of these phases is changing. The diffractograms demonstrate that the amount of quartz in BA is higher compared to that of fresh fly ash, while the amount of mullite is lower compared to that of core borehole V3. At the same time, the background also changes, which is primarily related to the presence of Fe phases. XRD analysis confirmed the presence of iron in the form of magnetite and hematite. A more significant change in phase composition was recorded at the depth of 38 m. Calcite (CaCO_3_) occurred here, and there was a significant decrease in background in the interval 15–28 2Theta, which in turn agrees with the results of XRF analysis showing the lowest proportion of Si-Al components from all depths of the core borehole recorded at this very depth. Calcite detected in the sample S1_38m next to high-temperature phases is of secondary origin from the limestone subsoil.

### 3.9. SEM

**Figure 7:**
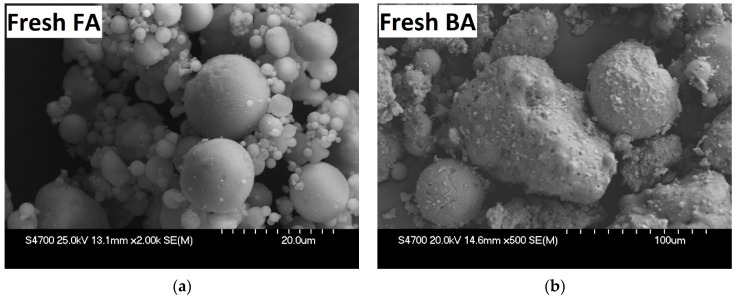
SEM Images of Fresh FA (**a**), magnified 2000× and Fresh BA (**b**), magnified 500×.

In Figure 7a below, SEM images are provided showing fresh FA with spherical particles and their typical diameter in micrometers. Figure 7b shows fresh bottom ash containing coarse, sintered particles of non-combustible coal components with a morphology resembling the grains of the original coal.

**Figure 8:**
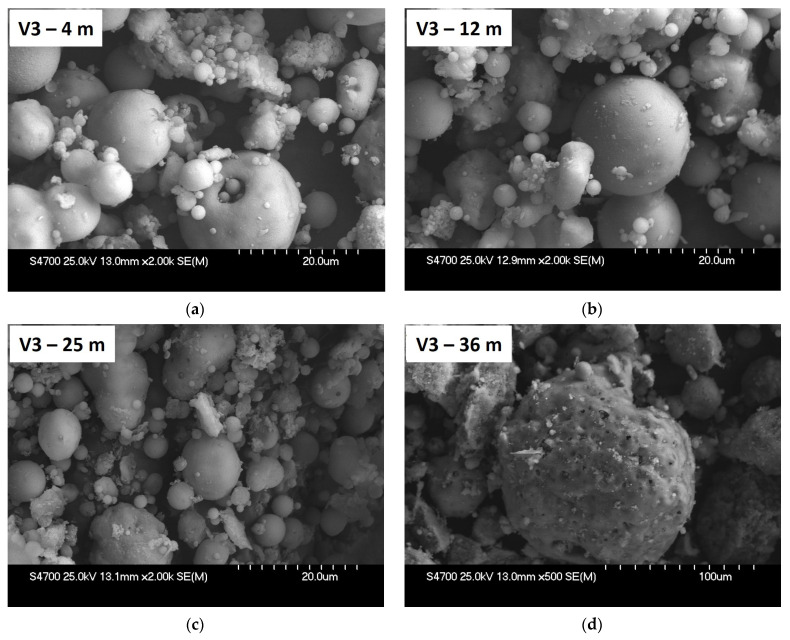
SEM Images of Agglomerates from Core Borehole V3 from Depths 4 m (**a**), 12 m (**b**), 25 m (**c**), magnified 2000× and 36 m (**d**), magnified 500×.

Figure 8 presents SEM images of core borehole V3 for the depths of 4, 12, 25 and 36 m. The morphology of predominantly spherical agglomerate particles at the depths of 4 and 12 m was found to be comparable to fresh fly ash from the Mělník power plant. Fresh ash and stockpiled ash from the depths of 4 and 12 m primarily contain spherical particles with a smooth surface. No significant changes were observed on the surface of spherical particles at the depth of 25 m, but there is a noticeable change in the fineness of extracted deposited ash V3, which corresponds to the results of granulometry and is probably related to ponding and separation of ponded ash. Significant changes in the morphology of the particles are evident at the depth of 36 m with noticeably coarser particles mostly of an irregular shape. These particles were not necessarily formed by agglomeration of the original fly ash, but they may have already been contained in the stockpiled material. Particles from a depth of 36 m and more correspond to the morphology of the bottom ash, and, at the same time, their appearance can be caused by corrosion and leaching, as also claimed in the work by Praharaj et al. [24]. The formation of secondary mineral phases can play a certain role here as core borehole.

**Figure 9:**
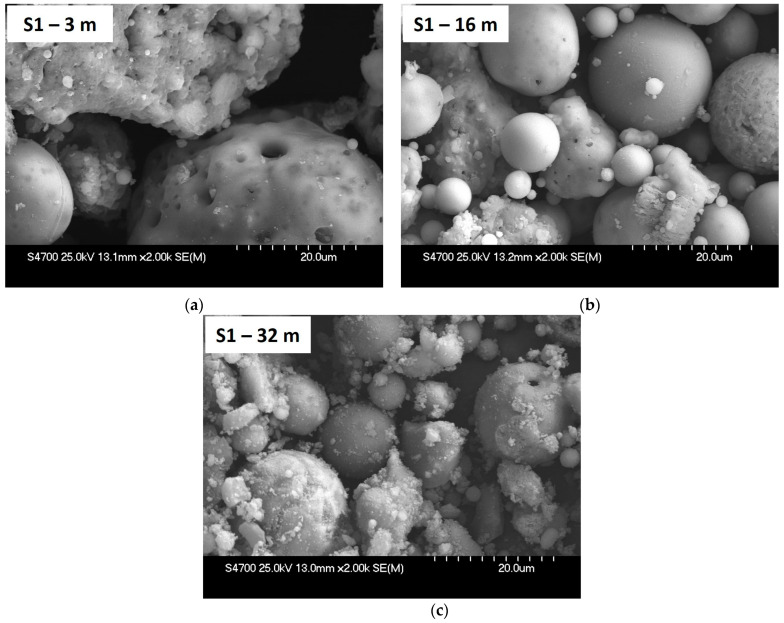
SEM Images of Deposited S1 Bottom Ash from Core Borehole Depths of 3 m (**a**), 16 m (**b**) and 32 m (**c**), magnified 2000×.

Figure 9 shows SEM images of deposited bottom ash from core borehole S1 for depths of 3 m, 16 m and 32 m. With increasing depth of the core borehole, disintegration of particles is evident as confirmed by granulometric curves for individual depths of core borehole S1. From the SEM images, it was found that the particles from core borehole S1 had a very similar appearance comparable to the original grains of fresh BA. Simultaneously, the etched and corroded surface of the particles can be observed, which corresponds to the conclusions drawn from the analysis of the leachates.

## 4. Conclusions

The main findings of the presented results can be summarized as follows:

- The obtained results from deposited and bottom ash in the locality Panský les of the Mělník power plant show that this material has basic chemical and physical properties comparable to fresh FA, resp. to fresh BA.
- It was proved that no significant chemical changes of the material (XRF, XRD) occurred due to hydrogeological phenomena across the entire stockpile. The conducted measurements of chemical properties proved the chemical stability of the material even after several decades of storage in the stockpile.
- The largest changes were evident in the analyses related to the leachability of SO_3_, Cl^−^ and F^−^. The pH values have not changed significantly, and the overall composition is pH neutral or alkaline.
- The processed analyses (SEM) do not show any significant formation of products of chemical reactions on the surface of individual particles. In the case of bottom ash, the disintegration of particles was recorded (PSD, SEM), growing with the increasing depth of the core borehole.
- When compared to the deposited agglomerate, fly ash for concrete (ČSN EN 450-1) is a finer material in terms of the particle size. For the following valuable use of the agglomerate, its further mechanical treatment by grinding and sorting is expected in the future.
- The storage concept for deposited ash has been demonstrated to be suitable for storing the ash meant for later processing.

## Figures and Tables

**Table 1:** Values of Specific Density and Specific Surface for Selected Samples from Core Boreholes V3 and S1 and for Fresh Fly Ash (FA) and Fresh Bottom Ash (BA).

Sample	Specific Density [kg/m^3^]	Specific Surface [m^2^/kg]
**FA fresh**	**2296 ± 6**	**334 ± 1**
**V3-4**	2121 ± 7	213 ± 1
**V3-8**	2113 ± 3	171 ± 2
**V3-12**	2152 ± 4	196 ± 2
**V3-16**	2054 ± 10	208 ± 1
**V3-20**	2095 ± 8	194 ± 1
**V3-25**	217 ± 5	381 ± 0
**V3-28**	2332 ± 4	112 ± 1
**V3-33**	2167 ± 7	145 ± 1
**V3-36**	2204 ± 9	138 ± 1
**V3-45**	2305 ± 4	247 ± 1
**BA fresh**	**2349 ± 7**	**218 ± 2**
**S1-3**	2097 ± 7	137 ± 3
**S1-6**	2035 ± 7	153 ± 3
**S1-10**	2209 ± 5	174 ± 2
**S1-16**	2098 ± 10	151 ± 2
**S1-22**	2109 ± 5	240 ± 4
**S1-26**	2335 ± 5	169 ± 2
**S1-32**	2290 ± 8	185 ± 1
**S1-38**	2219 ± 1	235 ± 4

**Table 2:** Oxide Composition of Fresh FA, Bottom Ash (BA) and Selected Deposit Samples from Core Boreholes V3 and S1 in locality Panský les by XRF analysis; measurement errors were below 1 wt.%.

Sample	Weight [%]																									
	SiO_2_	Al_2_O_3_	Fe_2_O_3_	Na_2_O	K_2_O	CaO	MgO	TiO_2_	P_2_O_5_	SO_3_	S^−2^	V_2_O_5_	Cr_2_O_3_	MnO	Co_3_O_4_	NiO	CuO	ZnO	SrO	ZrO_2_	Nb_2_O_5_	BaO	CeO_2_	As_2_O_3_	Others	LOI
FA fresh	47.19	34.29	7.52	0.47	1.19	1.86	0.97	3.10	0.26	0.54	0.00	0.08	0.02	0.05	0.01	0.01	0.02	0.03	0.05	0.06	0.02	0.08	0.01	0.01	0.04	2.10
**V3-4**	49.28	35.24	6.12	0.37	1.01	1.89	0.93	2.56	0.16	0.36	0.00	0.07	0.03	0.04	0.01	0.02	0.01	0.02	0.03	0.06	0.02	0.04	0.02	0.00	0.01	1.70
**V3-8**	48.27	34.75	6.98	0.44	0.99	1.95	0.87	2.42	0.15	0.40	0.00	0.07	0.03	0.05	0.01	0.01	0.01	0.02	0.03	0.05	0.01	0.05	0.00	0.00	0.08	2.35
**V3-12**	49.20	33.92	7.61	0.38	1.24	1.86	0.96	2.11	0.18	0.38	0.00	0.06	0.02	0.05	0.01	0.02	0.01	0.02	0.04	0.05	0.01	0.05	0.00	0.00	0.06	1.76
**V3-16**	50.19	33.66	6.56	0.46	1.42	1.89	1.00	2.17	0.18	0.37	0.00	0.07	0.02	0.04	0.01	0.01	0.01	0.02	0.04	0.04	0.01	0.06	0.00	0.01	0.05	1.68
**V3-20**	49.78	34.55	6.07	0.36	1.26	1.77	0.91	2.35	0.18	0.32	0.00	0.07	0.03	0.04	0.01	0.01	0.01	0.02	0.04	0.05	0.01	0.05	0.01	0.00	0.04	2.05
**V3-25**	49.30	33.09	5.74	0.44	1.60	2.78	1.09	1.98	0.21	0.13	0.00	0.07	0.02	0.04	0.01	0.01	0.02	0.04	0.04	0.04	0.01	0.06	0.01	0.01	0.06	3.19
**V3-28**	47.66	30.91	4.11	0.22	1.64	2.10	0.86	1.31	0.12	0.00	0.23	0.04	0.02	0.02	0.01	0.00	0.00	0.00	0.03	0.03	0.01	0.03	0.01	0.00	0.06	10.60
**V3-33**	49.75	32.15	6.20	0.40	1.36	2.29	1.09	2.23	0.21	0.21	0.00	0.06	0.02	0.05	0.01	0.01	0.01	0.01	0.04	0.05	0.01	0.08	0.00	0.00	0.04	3.71
**V3-36**	49.20	30.53	5.24	0.31	1.52	1.93	1.03	2.03	0.17	0.00	0.14	0.06	0.02	0.04	0.00	0.01	0.01	0.01	0.03	0.04	0.01	0.04	0.01	0.00	0.03	7.58
**V3-45**	50.94	31.66	6.72	0.42	1.44	1.92	1.10	2.74	0.20	0.00	0.02	0.07	0.02	0.05	0.01	0.01	0.01	0.03	0.04	0.06	0.02	0.07	0.02	0.01	0.04	2.38
**BA fresh**	**44.25**	**32.23**	**8.12**	**0.30**	**0.91**	**1.57**	**0.67**	**5.12**	**0.28**	**0.00**	**0.29**	**0.09**	**0.02**	**0.11**	**0.01**	**0.00**	**0.02**	**0.01**	**0.05**	**0.08**	**0.03**	**0.07**	**0.03**	**0.00**	**0.04**	**5.70**
**S1-3**	47.68	31.49	3.79	0.26	1.56	1.71	0.82	1.80	0.13	0.00	0.21	0.06	0.02	0.02	0.00	0.01	0.01	0.00	0.03	0.04	0.01	0.05	0.00	0.00	0.04	10.25
**S1-6**	51.51	33.74	5.53	0.26	1.53	1.64	0.95	1.81	0.13	0.00	0.06	0.06	0.02	0.03	0.01	0.00	0.01	0.01	0.03	0.04	0.01	0.04	0.01	0.00	0.04	2.54
**S1-10**	50.01	32.16	7.22	0.38	1.34	2.08	1.04	1.99	0.15	0.00	0.08	0.06	0.02	0.06	0.00	0.00	0.01	0.01	0.03	0.04	0.01	0.06	0.00	0.00	0.06	3.18
**S1-16**	52.11	31.89	6.64	0.38	1.31	2.23	0.97	2.52	0.18	0.00	0.01	0.07	0.03	0.05	0.01	0.01	0.01	0.01	0.04	0.05	0.02	0.05	0.01	0.00	0.04	1.37
**S1-22**	51.39	32.06	6.06	0.36	1.51	1.79	1.03	2.13	0.16	0.00	0.08	0.06	0.02	0.04	0.00	0.01	0.01	0.02	0.03	0.04	0.01	0.06	0.02	0.00	0.04	3.05
**S1-26**	51.44	31.38	6.60	0.39	1.42	1.90	1.06	2.47	0.18	0.00	0.05	0.06	0.02	0.05	0.00	0.00	0.01	0.01	0.04	0.05	0.01	0.05	0.02	0.00	0.05	2.72
**S1-32**	49.07	30.75	7.49	0.41	1.14	2.22	0.98	3.09	0.22	0.00	0.07	0.07	0.02	0.07	0.01	0.01	0.02	0.02	0.04	0.05	0.02	0.08	0.00	0.01	0.03	4.12
**S1-38**	41.88	25.25	6.15	0.25	1.01	11.96	0.90	2.75	0.18	0.70	0.00	0.07	0.02	0.06	0.01	0.01	0.01	0.02	0.05	0.05	0.01	0.06	0.00	0.01	0.04	8.57

**Table 3:** Values of pH, Electrical Conductivity (EC) and Total Dissolved Solids (TDS) for Fresh FA, Bottom Ash BA and Samples from Core Boreholes V3 and S1; measurement errors were below 1%.

	pH	EC [mS.m^−1^]	TDS [mg.L^−1^]
**FA fresh**	**8.23**	**241.0**	**2220**
**V3-4**	7.71	55.9	392
**V3-8**	7.41	61.1	411
**V3-12**	7.39	56.4	370
**V3-16**	6.84	70.1	442
**V3-20**	7.31	58.9	366
**V3-25**	7.54	45.1	288
**V3-28**	7.90	33.2	189
**V3-33**	7.81	22.6	123
**V3-36**	7.55	14.0	<100
**V3-45**	7.69	14.0	<100
**BA fresh**	**7.38**	**241.0**	**2430**
**S1-3**	6.96	16.8	111
**S1-6**	6.92	10.1	<100
**S1-10**	7.28	14.6	<100
**S1-16**	7.66	15.1	<100
**S1-22**	7.23	16.1	<100
**S1-26**	7.38	12.8	122
**S1-32**	7.68	13.7	<100
**S1-38**	10.39	56.7	388

**Table 4:** Leachability Analysis of Trace Elements in Fresh FA, BA and in Samples from Core Boreholes V3 and S1. Measurement errors were below 1 wt.%.

	Leach Analysis [mg/L]												
	As	Ba	B	Al	Cd	Cr	Mo	Ni	Se	V	Cl^−^	F^−^	SO3^2−^
**ASVEP**	**0.010**	**0.050**	**0.300**	**0.200**	**0.00050**	**0.0500**	**0.0050**	**0.0200**	**0.010**	**0.018**	*	*	*
FA fresh	**0.036**	**0.066**	**1.060**	**0.291**	<0.00050	0.0180	**0.0450**	<0.0050	**0.013**	**0.061**	25.10	1.97	1510
V3-4	**0.025**	**0.053**	**0.748**	0.090	<0.00050	<0.0050	**0.0373**	<0.0050	**0.023**	**0.038**	<5.00	1.96	240
V3-8	**0.010**	**0.067**	**1.860**	0.113	<0.00050	<0.0050	**0.0467**	0.0058	**0.028**	**0.021**	30.30	1.55	242
V3-12	0.007	**0.054**	**0.860**	0.064	**0.00128**	<0.0050	**0.0336**	0.0061	<0.010	0.013	17.40	1.23	229
V3-16	**0.051**	**0.083**	**1.080**	0.028	<0.00050	<0.0050	**0.0637**	0.0071	**0.029**	**0.040**	27.40	1.99	301
V3-20	**0.032**	**0.076**	**0.765**	0.042	<0.00050	<0.0050	**0.0604**	<0.0050	**0.028**	**0.029**	20.00	1.95	237
V3-25	**0.100**	**0.059**	0.273	0.080	<0.00050	<0.0050	**0.0098**	<0.0050	**0.041**	**0.059**	20.70	1.12	150
V3-28	**0.017**	<0.050	0.221	0.110	<0.00050	<0.0050	<0.0050	<0.0050	**0.022**	**0.036**	<5.00	1.05	68
V3-33	**0.046**	**0.050**	0.226	0.163	<0.00050	<0.0050	**0.0090**	<0.0050	**0.038**	**0.058**	<5.00	<1.00	50
V3-36	**0.103**	<0.050	0.245	0.078	<0.00050	<0.0050	**0.0091**	<0.0050	**0.037**	**0.101**	5.16	<1.00	21
V3-45	**0.210**	<0.050	0.181	0.103	<0.00050	<0.0050	**0.0084**	<0.0050	**0.037**	**0.101**	5.11	<1.00	18
BA fresh	**0.066**	**0.070**	**1.850**	0.177	<0.00050	<0.00050	**0.0600**	<0.0050	**0.025**	**0.048**	26.80	0.00	1570
S1-3	<0.005	<0.050	**0.577**	<0.025	<0.00050	<0.0050	**0.0056**	<0.0050	<0.010	**0.028**	<5.00	<1.00	49
S1-6	0.009	<0.050	0.198	0.030	<0.00050	<0.0050	<0.0050	<0.0050	<0.010	0.008	<5.00	<1.00	27
S1-10	**0.012**	<0.050	0.259	0.037	<0.00050	<0.0050	<0.0050	<0.0050	**0.010**	**0.022**	<5.00	<1.00	39
S1-16	**0.147**	<0.050	0.225	**0.218**	<0.00050	<0.0050	**0.0074**	<0.0050	**0.034**	**0.096**	<5.00	1.10	22
S1-22	**0.058**	<0.050	**0.324**	0.032	<0.00050	<0.0050	**0.0592**	<0.0050	**0.020**	**0.061**	<5.00	1.00	42
S1-26	**0.046**	<0.050	0.272	0.035	<0.00050	<0.0050	**0.0054**	<0.0050	**0.021**	**0.042**	<5.00	<1.00	26
S1-32	**0.097**	<0.050	0.193	0.096	<0.00050	<0.0050	**0.0064**	<0.0050	**0.028**	**0.077**	<5.00	1.05	24
S1-38	**0.039**	<0.050	**0.475**	1.320	<0.00050	0.0181	**0.0273**	<0.0050	**0.027**	**0.214**	<5.00	<1.00	192

* Not specified.

## Data Availability

Not applicable.
